# Assessment of depression severity with the PHQ-9 in cancer patients and in the general population

**DOI:** 10.1186/s12888-016-0728-6

**Published:** 2016-02-02

**Authors:** Andreas Hinz, Anja Mehnert, Rüya-Daniela Kocalevent, Elmar Brähler, Thomas Forkmann, Susanne Singer, Thomas Schulte

**Affiliations:** Department of Medical Psychology and Medical Sociology, University of Leipzig, Leipzig, Germany; Section of Psychooncology, Department of Medical Psychology and Medical Sociology, University of Leipzig, Leipzig, Germany; Institute and Policlinic for Medical Psychology, University Medical Center Hamburg-Eppendorf, Hamburg, Germany; Department of Psychosomatic Medicine and Psychotherapy, University of Mainz, Mainz, Germany; Institute of Medical Psychology and Medical Sociology, University Hospital of RWTH Aachen, Aachen, Germany; Department of Medical Biostatistics, Epidemiology, and Informatics, University of Mainz, Mainz, Germany; Rehabilitation Clinic Bad Oexen, Bad Oeynhausen, Germany

**Keywords:** Depression, Cancer, Depressive symptoms, Psychometrics

## Abstract

**Background:**

The Patient Health Questionnaire PHQ-9 is a widely used instrument to screen for depression in clinical research. The first aim of this study was to psychometrically test the PHQ-9 in a large sample of cancer patients. The second aim was to calculate unbiased estimates of the depression burden for several cancer groups taking into account age and gender distributions.

**Methods:**

A sample of 2,059 cancer patients with varying diagnoses were examined in this study six months after discharge from a rehabilitation clinic. A representative sample of 2,693 people from the general population served as controls. Expected PHQ-9 mean scores of the general population sample, regressed on age and gender, were calculated to enable a fair comparison of different groups of cancer patients.

**Results:**

While the reliability (Cronbach’s alpha) for the PHQ-9 scale was good (alpha ≥ 0.84), the CFA fit indices of the one-dimensional solution were unsatisfactory in the patients’ sample. The factorial analysis confirmed two factors. PHQ-9 mean scores for 15 types of cancer are given, ranging from 4.0 (prostate) to 8.2 (thyroid gland). Differences between expected mean scores (derived from the general population) and raw mean scores of the cancer subsamples are reported that provide a better estimate of the depression burden.

**Conclusions:**

The results confirmed that the PHQ-9 performs well in testing depression in cancer patients. Regression coefficients can be used for performing unbiased comparisons among cancer groups, not only for this study. The burden of patients with testis cancer and Hodgkin lymphoma is underestimated when age and gender are not taken into account.

## Background

Depression is frequently observed in cancer patients. Meta-analyses reported a 25 % prevalence of all types of depression among cancer patients [1] and a 32 % prevalence of mental health conditions in general [2]. Depression may negatively affect treatment outcomes [3] and can be associated with elevated mortality in cancer patients [4].

Oncologists often fail to detect depression in their patients [5, 6]. Therefore, it is important to use standardized and easily applicable tools to detect depression. There are several screening instruments that proved to be effective for that purpose. The most often used questionnaires measuring depression in cancer patients are the Hospital Anxiety and Depression Scale HADS [7], the Beck Depression Inventory BDI [8] and the Center for Epidemiologic Studies Depression Scale CES-D [9], for a new summarizing review cf. [10]. A further, freely available and more recently developed questionnaire is the Patient Health Questionnaire PHQ-9 [11]. Its validity has been proven in several studies [12–15]. Normative scores are available [16], and two studies supply tools for converting scores between PHQ-9 and other depression scales [17, 18]. The PHQ-9 is generally used in its original one-dimensional form (sum score of the 9 items), but several psychometric studies with multiple disease groups challenged the one-dimensional solution [19, 20] and showed that two-dimensional solutions fitted better [21–25]. The assignments of the items to these two factors were not totally identical in these studies, but all of them obtained one factor concentrating on emotional and cognitive aspects (depressed mood, feeling worthless, and thoughts of death), and the other on somatic aspects (sleep problems, loss of energy, and appetite problems). The first central aim of this study was to test whether such a two-dimensional solution could also be found in a sample of cancer patients. In particular, we test the specific two-dimensional model that performed best in three [22, 23, 25] of the five [21–25] studies. In addition, the psychometric properties of the items in terms of item-test correlations and the correlations with other scales on emotional and somatic factors were to be examined.

Furthermore, there are age and gender differences influencing the PHQ-9 scores in the general population [16]. These differences should be taken into account when comparing patients with different cancer locations. It has often been documented that depression is relatively high in breast cancer patients [26] and low in prostate cancer patients [27]. However, to what degree is this difference due to different age and gender distributions? Unbiased comparisons between cancer groups can be done by calculating expected mean scores from the general population using linear regression analyses, cf. [28], and by considering the differences between the patients’ group means and these expected mean scores derived from the general population. The second objective of this study was to perform such regression analyses and compare the mean depression levels for multiple cancer types with and without correction for age and gender effects.

In summary, the aims of this paper were

- to test psychometric properties and the factorial structure of the PHQ-9,
- to calculate a regression analysis for the assessment of expected mean scores that help evaluate the PHQ-9 mean values of different cancer entities, and
- to calculate unbiased estimates of the depression burden for several cancer diagnoses.

## Methods

### Cancer patients

Between 2011 and 2012, a group of 3,592 consecutive patients treated in a German rehabilitation clinic were asked to participate in the study. In Germany, most cancer patients are offered the opportunity to participate in rehabilitation program to regain physical and psychosocial functioning. During that program, generally lasting three weeks, the patients receive physiotherapy, physical fitness exercises, relaxation techniques, counseling concerning nutritional and occupational issues, and coping training. Inclusion criteria were age 18 years and above, absence of severe cognitive impairment, and sufficient command of the German language. Written informed consent was obtained from the study participants after full explanation of the purpose and nature of the data collection and storage. This research meets the ethics guidelines of the institution where the study was performed, including adherence to the legal requirements of Germany. A total of 2909 (81.0 %) of the 3,592 candidates agreed to participate in the study. These patients were sent a letter with several questionnaires six months after being discharged from the rehabilitation clinic. In all, 2059 patients responded (57.3 % of all patients; 72.4 % of the patients receiving the letter). Table 1 presents characteristics of the sample.

**Table 1:** Sociodemographic characteristics of the patients’ sample

	Total (*N* = 2,059)		Males (*N* = 1,210)		Females (*N* = 849)	
	*N*	%	*N*	%	*N*	%
Age Mean (SD) in years	62.4	(14.2)	64.8	(11.9)	59.1	(16.4)
Age category						
18-35 y.	141	6.8	53	4.4	88	10.4
36-45 y.	138	6.7	30	2.5	108	12.7
46-55 y.	233	11.3	108	8.9	125	14.7
56-65 y.	498	24.2	333	27.5	165	19.4
66-75 y.	788	38.3	544	45.0	244	28.7
76-94 y.	261	12.7	142	11.7	119	14.0
Diagnosis						
Prostate	640	31.1	640	52.9	0	0.0
Breast	346	16.8	3	0.2	343	40.4
Colon	196	9.5	91	7.5	105	12.4
Kidney	119	5.8	68	5.6	51	6.0
Bladder	91	4.4	71	5.9	20	2.4
Rectum	90	4.4	56	4.6	34	4.0
Hodgkin lymphoma	64	3.1	28	2.3	36	4.2
Stomach	53	2.6	35	2.9	18	2.1
Ovary	41	2.0	0	0.0	41	4.8
Testis	35	1.7	35	2.9	0	0.0
Other non-Hodgkin lymphoma	31	1.5	17	1.4	14	1.6
Pancreas	31	1.5	21	1.7	10	1.2
Oesophagus	30	1.5	26	2.1	4	0.5
Non-follicular lymphoma	30	1.5	16	1.3	14	1.6
Thyroid gland	29	1.4	8	0.7	21	2.5
Others	233	11.3	95	7.9	138	16.3
Number of tumors						
1	1,851	89.9	1,084	89.6	767	90.3
≥2	208	10.1	126	10.4	82	9.7
Surgery						
no	208	10.1	110	9.1	98	11.5
yes	1,851	89.9	1,100	90.9	751	88.5
Radiation						
no	1,424	69.2	973	80.4	451	53.1
yes	635	30.8	237	19.6	398	46.9
Chemotherapy						
no	1,326	64.4	908	75.0	418	49.2
yes	733	35.6	302	25.0	431	50.8
Endocrine therapy						
no	1,782	86.5	1,142	94.4	640	75.4
yes	277	13.5	68	5.6	209	24.6
Antibody therapy						
no	1,934	93.9	1,166	96.4	768	90.5
yes	125	6.1	44	3.6	81	9.5

### General population

The data basis for the control group was a survey of the German general population (age 14 to 92 years), conducted in two waves: in 2003 (*n* = 2,500) and in 2008 (*n* = 2,518). Age, gender, and regional distribution were the major criteria for representativeness. The random-route procedure included random selection of sample points within Germany, random selection of houses and household within these areas, and random selection of the target person within the household. The summarized response rate was 63 %; 5,018 subjects (54 % females) participated in the study. Written informed consent was obtained by all study participants. In order to allow for a fair comparison with the patient sample, we selected a subsample so that the age and gender distribution matched that of the patients. The final sample of the general population comprised 2,693 subjects between 40 and 92 years, mean age: 62.3 (*SD* = 11.3) years; 1,579 males (59 %) and 1,114 females (41 %), in accordance with the distribution of the patients’ sample. The aims of the general population studies were to obtain normative values for several questionnaires. Further details of the studies have been reported elsewhere [16]. The study was approved by the Ethics Committee of the University of Leipzig, Germany.

### Instruments

#### PHQ-9

The PHQ-9 is a screening instrument with 9 items (see Table 2), developed to measure depression. For each item the patients are asked to assess how much they were bothered by the symptoms over the last two weeks. There are four answer options: not at all (0), several days (1), more than half of the days (2), and nearly every day (3). The sum score (range 0 to 27) indicates the degree of depression, with scores of ≥5, ≥10, and ≥15 representing mild, moderate, and severe levels of depression [11]. While the PHQ-9 was used in both the patients’ and the general population group, the further questionnaires (see below) were only administered to the patients’ group.

**Table 2:** Mean scores of the PHQ-9 items and the sum score for cancer patients and the general population

Item		Patients		General population		*d*
		*M*	*SD*	*M*	*SD*	
1	Loss of interest	0.71	0.73	0.52	0.63	0.30
2	Feeling depressed	0.59	0.70	0.38	0.60	0.35
3	Sleep problems	1.07	0.95	0.63	0.75	0.59
4	Loss of energy	0.97	0.78	0.65	0.70	0.46
5	Appetite problems	0.45	0.75	0.28	0.56	0.30
6	Self-blame	0.33	0.61	0.20	0.48	0.27
7	Concentration problems	0.64	0.71	0.39	0.60	0.42
8	Agitation/retardation	0.35	0.64	0.16	0.45	0.42
9	Suicidal ideation	0.15	0.43	0.10	0.35	0.14
	Sum score	5.26	4.27	3.30	3.65	0.54

*d*, effect size

#### EORTC QLQ-C30

The quality of life questionnaire EORTC QLQ-C30 [29] consists of 30 items and incorporates five functioning scales (physical, role, emotional, social, and cognitive), three symptom scales (fatigue, pain and nausea/vomiting), a global health status/QoL scale and six single items. Higher functioning scores represent better functioning/QoL, whereas higher symptom scores represent more severe symptoms.

#### FoP

The 12-item Fear of Progression Questionnaire is a short form of the original 43-item Fear of Progression instrument [30] designed to assess fear of cancer progression. The items are scored on a five-point Likert scale, ranging from 1 (‘never’) to 5 (‘very often’), resulting in a sum score from 12 to 60.

#### GAD-2

The Generalized Anxiety Disorder Questionnaire GAD-2 is a 2-item short form of the GAD-7 [31]. Together with the PHQ-2, the GAD-2 forms the PHQ-4 [32]. The answer options are identical to those of the PHQ-9.

### Statistical analyses

Means, standard deviations, reliability coefficients (Cronbach’s alpha) and part-whole-corrected item-test-correlations were calculated. Mean score differences were expressed in terms of effect sizes *d* according to Cohen [33]. Principal component analyses (PCA) with two factors and varimax rotation were performed since previous studies also detected two-dimensional structures [21, 22]. Confirmatory factorial analyses (CFA) [34] were performed with Mplus. We used the following criteria: Bayesian Information Criterion (BIC), Standardized Root Mean Square Residual (SRMR), Root Mean Square Error of Approximation (RMSEA) [35], Comparative Fit Index (CFI) [36], and Tucker Lewis Index (TLI) [37]. RMSEA should be lower than 0.10, and CFI and TLI should be greater than or equal to 0.95 [34].

A linear regression analysis of the general population’s sample was performed, with age and gender as independent variables and the PHQ-9 sum score as the dependent variable. Expected PHQ-9 mean scores were calculated with these regression coefficients for several cancer groups, depending on their age and gender distributions. These expected mean scores were then compared with the raw mean scores of the cancer groups.

## Results

### Mean scores of the PHQ-9 items

The mean sum score of the PHQ-9 in the patients’ sample was 5.26 (Table 2). According to the cut-off criteria for mild (5–9), moderate (10–14), and severe (≥15) depression, the frequencies were 49.8 % (no), 35.1 % (mild), 11.3 % (moderate), and 3.7 % (severe) depression for the patients. In the general population, the percentages for no, mild, moderate, and severe depression were 72.2 %, 21.2 %, 5.1 %, and 1.5 %, respectively.

On the item level, the mean scores (Table 2) of the cancer sample ranged from 0.15 (suicidal ideation) to 1.07 (sleep problems). All items showed higher mean scores in the cancer group compared with the general population. The greatest differences between both samples, expressed in terms of effect sizes (*d* > 0.40), were found for items 3 (sleep problems), 4 (loss of energy), 7 (concentration problems), and 8 (psychomotor agitation/retardation).

### Reliability and factorial analyses

The reliability coefficient (Cronbach’s alpha) of the PHQ-9 for the patients’ sample was 0.84. The highest part-whole-corrected correlations between item and sum score (*r*_it_) were obtained for items 4 (loss of energy), 2 (feeling depressed), 7 (concentration problems), and 1 (loss of interest) (Table 3). All items contributed positively to the reliability of the scale. The contribution of the last item (suicidal ideation) was lowest, but positive nevertheless.

**Table 3:** Factor loadings and item-test correlations for the cancer patients

Item		F1	F2	*r*_*it*_	alpha (del)
1	Loss of interest	.54	.47	.**60**	.82
2	Feeling depressed	.49	.**66**	.**68**	.82
3	Sleep problems	.**75**	.03	.51	.84
4	Loss of energy	.**78**	.27	.**70**	.81
5	Appetite problems	.58	.27	.52	.83
6	Self-blame	.33	.**65**	.54	.83
7	Concentration problems	.**65**	.32	.**61**	.82
8	Agitation/retardation	.**63**	.21	.52	.83
9	Suicidal ideation	.03	.**85**	.43	.84

*F*1, *F*2 factors of the principal component analysis, *r*_*it*_ part-whole corrected item-test correlation, alpha (del) cronbach’s alpha if item deleted; bold: coefficients ≥ .60

Results of the 2-factorial principal components analysis (PCA) for the patients’ sample are also given in Table 3. The theoretically assumed structure (Items 1, 2, 6, and 9 in one factor and items 3, 4, 5, 7, and 8 in the other) was realized with one exception (item1: loss of interest). CFA results for the total scale and for the two-factorial structure are given in Table 4, indicating a better fit for the latter model.

**Table 4:** CFA results for the cancer patients

	Chi^2^	BIC	SRMR	RMSEA	CFI	TLI
1-factorial model	494.1	32271.2	0.041	0.094	0.915	0.887
2-factorial model	302.0	32087.4	0.034	0.074	0.950	0.931

### Relationship between PHQ-9 scores and other scales

The PHQ-9 items were correlated with several scales of other questionnaires (Table 5). In the left part of Table 5, the scales focus the affective and mental component, while in the right part the scales also include physical aspects.

**Table 5:** Correlations between item scores of the PHQ-9 and scale scores of other instruments in the cancer patients’ sample

Item		EORTC	EORTC	GAD	FoP	EORTC	EORTC	EORTC
		Emot. Funct.	Cognit. Funct.	Anxiety	Fear of Progression	Quality of Life	Physical Funct.	Fatigue
1	Loss of interest	-.**51**	-.37	.45	.43	-.**53**	-.43	.49
2	Feeling depressed	-.**62**	-.39	.**60**	.**54**	-.**51**	-.40	.49
3	Sleep problems	-.44	-.33	.40	.41	-.38	-.34	.46
4	Loss of energy	-.**61**	-.48	.**52**	.**52**	-.**58**	-.**51**	.**70**
5	Appetite problems	-.42	-.32	.39	.37	-.39	-.34	.43
6	Self-blame	-.49	-.36	.49	.40	-.32	-.22	.34
7	Concentration problems	-.53	-.**72**	.48	.44	-.42	-.36	.49
8	Agitation/retardation	-.46	-.48	.47	.36	-.35	-.35	.42
9	Suicidal ideation	-.37	-.30	.47	.32	-.29	-.24	.27
	Sum score	-.74	-.62	.70	.63	-.63	-.54	.69

*Bold* coefficients > 0.50. All coefficients are statistically significant with *p* < 0.001

There is a clear correspondence between item 7 (concentration problems) and Cognitive functioning (*r* = −0.72). Item 4 (loss of energy) is highly correlated with all scales, including affective scales and those with physical aspects. Among the four items (1, 2, 6, 9) assigned to Factor 2 (emotional and cognitive aspect), all correlations with the Emotional functioning scale are higher than those with the EORTC fatigue scale. On the other hand, among the five items (3, 4, 5, 7, 8) of Factor 1 (somatic aspect), only three items (3, 4, and 5) showed higher correlations with fatigue, compared with the correlations to Emotional functioning.

### Tumor-specific analyses

Table 6 shows PHQ-9 mean scores for all tumor sites with subsample sizes of 25 and above, arranged according to the PHQ-9 mean score. There are great differences among the subsamples concerning age and sex distribution. Since the PHQ-9 mean scores depend on age and sex in the general population, a fair comparison among the tumor sites requires the consideration of these age and sex differences.

**Table 6:** PHQ-9 scores, broken down by cancer site

	*n*	PHQ-9 Mean	% Females	Mean age	Expected PHQ-9 Mean	Difference
Thyroid gland	29	8.2	72	50.6	3.0	5.2
Other Non-Hodgkin lymphoma	31	7.6	45	57.2	3.1	4.5
Ovary	41	7.6	100	63.2	3.5	4.1
Oesophagus	30	6.9	13	64.0	3.3	3.6
Breast	346	6.3	99	57.0	3.3	3.0
Non-follicular lymphoma	30	6.1	47	61.4	3.3	2.8
Pancreas	31	5.9	32	65.2	3.4	2.5
Stomach	53	5.8	34	66.9	3.4	2.4
Hodgkin lymphoma	64	5.6	56	37.1	2.4	3.2
Rectum	90	5.3	38	65.8	3.4	1.9
Kidney	119	5.2	43	65.9	3.4	1.8
Colon	196	5.0	54	70.4	3.6	1.4
Testis	35	4.9	0	35.9	2.2	2.7
Bladder	91	4.6	22	69.5	3.5	1.1
Prostate	640	4.0	0	66.9	3.3	0.7
Total	2,059	5.26	41	62.3	3.3	2.0

Expected PHQ-9 mean: mean score of a subgroup of the general population with the same age and gender distribution as the patients’ sample; Difference: Difference between the PHQ-9 mean and the expected mean, indicating the burden of the disease

The linear regression analysis of the general population’s sample yielded the following regression equation:

$$ \mathrm{P}\mathrm{H}\mathrm{Q} = 0.0367\ *\ \mathrm{age} + 0.310\ *\ \mathrm{sex} + 0.884. $$

Sex is to be coded with the values of 0 (males) and 1 (females). For example, the expected PHQ-9 score of a 60-years old woman is 0.0367 * 60 + 0.310 * 1 + 0.884 = 3.396. For the whole sample of the general population (41 % women; mean age: 62.2 years), the calculation is as follows: PHQ-9 (expected) = 0.0367 * 62.2 + 0.310 * 0.41 + 0.884 = 3.294. The column “Expected PHQ-9 Mean” in Table 6 shows these expected values for samples with the age and gender distribution of the cancer groups. These expected means deliver the basis for the comparison of depression burden of the different cancer patients groups.

All groups of patients show higher mean values than the (matched) controls, with differences ranging from 0.7 (prostate) to 5.2 (thyroid gland). The sequence of the cancer sites according to the PHQ-9 mean scores is similar to the sequence according to the age-and gender-corrected mean values (right part of Table 6). Patients with testis cancer have a mean PHQ-9 score of 4.9, which is the third lowest mean value in Table 6. Taking into account that the patients are males and that they are relatively young, the difference between the actual score and the expected one (*diff* = 2.7) indicates a higher level of distress in this group. A similar phenomenon can be observed for patients with Hodgkin lymphoma.

## Discussion

The first aim of this study was to test the factorial structure of the PHQ-9 administered to cancer patients. The results of the factorial analyses demonstrate that a two-dimensional model according to [23] performed better than the one-dimensional model. With one exception (item 1; loss if interest) the hypothetically assumed structure emerged in the PCA. It is interesting to note that the two items that were selected for the PHQ-2 (loss of interest and feeling depressed) reached good part-whole corrected item-test-correlations (0.60 and 0.68), and that they had positive loadings in both factors in the PCA. Together with the results of other studies reported in the literature, we can conclude that the PHQ-9 comprises two aspects, an affective-cognitive component (feeling depressed, self-blame, and suicidal ideation) and a somatic component (sleep problems, loss of energy, and appetite problems), but that the assignment of the remaining three items to the scales according to the factorial analyses (loss of interest, concentration problems, and agitation/retardation) is less clear. The reliability coefficient of the total scale (Cronbach’s alpha) was good (alpha = 0.84), and all items contributed to this scale. This is similar to the results of other studies [38, 39]. Insufficient CFA fit indices for the total sum scale are also found in other depression questionnaires (e.g., [40]). As long as there is no other structure of the questionnaire that can be reliably replicated in several studies, we believe that it is best to maintain the sum score.

Sleep problems (item 3) and loss of energy (item 4) were the symptoms that differed most greatly between the cancer patients and the general population, followed by concentration problems (item 7) and agitation/retardation (item 8). As such, “classical” depression features like feeling depressed and loss of interest, were not reported to be key burdens of cancer patients half a year after rehabilitation. Item 8 contains two contradictory aspects of psychomotorics: agitation and retardation. This item fitted most poorly in the Forkmann et al. study [19] and was therefore excluded there. Clinicians report that patients have difficulties answering this item because of its seemingly contradictory nature. In the PCA, the item was associated with factor 1 in the patients’ sample. It cannot be clearly interpreted. Item 9 (suicidal ideation) showed very low mean scores, and the item-total correlations were lowest, though both coefficients were greater than 0.40. The contribution to the sum score of the PHQ-9 is small. However, physicians may obtain relevant information when this item is not totally denied [41]. Taking these properties of the PHQ-9 together, it is an advantage of this short instrument that it can nevertheless been used for different purposes: (a) general screening for depression, (b) focusing on two aspects of depression according to the two factors, and (c) considering single items such as suicidal ideation.

The mean score differences between cancer patients and the general population were most pronounced for the items indicating sleep problems and loss of energy. These items belong to Factor 1 and indicate general health problems. All nine items are heightened in the cancer patients’ sample, but it is worth noticing that the health-related components are most strongly affected.

The comparison between the cancer types confirmed high degrees of depression in patients with thyroid cancer [42] and low degrees in those with prostate cancer [27]. While breast cancer patients also show high mean levels of mental distress [43], in this study breast cancer was in the upper margin, but not at the top. Moreover, PHQ-9 mean scores were presented for several other, more seldom types of cancer that have not been extensively examined in psycho-oncological research. In addition to the raw PHQ-9 mean scores for the different cancer types, we also calculated the differences between these mean scores and the expected mean scores, based on the age and gender distribution. There were no great differences between raw scores and corrected scores in the sequences (Table 6). However, for cancer types with large proportions of males and young patients, (testis, Hodgkin lymphoma), the burden of cancer is underestimated when only simple mean scores are considered. Regression analyses such as those performed here can also be calculated for other questionnaires in order to provide a basis for unbiased comparisons among subgroups of patients.

Some *limitations* of this study should be mentioned. We examined patients half a year after discharge from a rehabilitation clinic. Patients with a very bad prognosis may be underrepresented or overrepresented in the sample. Though we believe that patients in a good health state are more compliant in filling in the questionnaire, resulting in a slight underestimation of the depression burden in the sample, we have no information on the non-participants. A further limitation is the limited information surrounding the health status of the respondents. In addition, participants of a rehabilitation program are not totally representative of all cancer patients. We only calculated CFA analyses for the one-dimensional model and one two-dimensional model. It would be possible to refine the CFA models and to arrive at better fit indices if several modifications were made such as: considering sub-dimensions, correlated error terms or removing items. However, special modifications, adapted to each data set, would not lead to generalizable results. Some patient groups in our study had small sample sizes, their depression mean scores should be considered with caution. Finally, the PHQ-9 is an economic screening instrument, which, however, is not a sufficient substitute for a clinical diagnosis of depression. Nevertheless, it can help provide aggregated information on the burden of special disease groups such as cancer patients.

## Conclusions

The results showed that the PHQ-9 is comprised of items that measure several aspects of depression, but that it is nevertheless useful to maintain the PHQ-9 as a one-dimensional scale in practical applications. The regression coefficients can be utilized to qualify the comparison among different groups of patients.

## Abbreviations

BDI: Beck Depression Inventory
CES-D: Center for Epidemiologic Studies Depression Scale
CFA: confirmatory factorial analysis
EORTC QLQ-C30: European Organization for Research and Treatment of Cancer Quality of Life Questionnaire C30
FoP: Fear of Progression questionnaire
GAD-2: General Anxiety Disorder Questionnaire-2
HADS: Hospital Anxiety and Depression Scale
PCA: principal component analysis
PHQ-9: Patient Health Questionnaire-9

## References

[1] Mitchell AJ (2007). Pooled results from 38 analyses of the accuracy of distress thermometer and other ultra-short methods of detecting cancer-related mood disorders. J Clin Oncol.

[2] Singer S, Das-Munshi J, Brähler E (2010). Prevalence of mental health conditions in cancer patients in acute care–a meta-analysis. Ann Oncol.

[3] Pirl WF, Greer JA, Traeger L, Jackson V, Lennes IT, Gallagher ER (2012). Depression and survival in metastatic non-small-cell lung cancer: Effects of early palliative care. J Clin Oncol.

[4] Pinquart M, Duberstein PR (2010). Depression and cancer mortality: a meta-analysis. Psychol Med.

[5] Passik SD, Dugan W, McDonald MV, Rosenfeld B, Theobald DE, Edgerton S (1998). Oncologists’ recognition of depression in their patients with cancer. J Clin Oncol.

[6] Singer S, Brown A, Einenkel J, Hauss J, Hinz A, Klein A (2011). Identifying tumor patients’ depression. Support Care Cancer.

[7] Zigmond AS, Snaith RP (1983). The Hospital Anxiety and Depression Scale. Acta Psychiatr Scand.

[8] Beck AT, Erbaugh J, Ward CH, Mock J, Mendelsohn M (1961). An inventory for measuring depression. Arch Gen Psychiatry.

[9] Radloff LS (1977). The Center for Epidemiologic Studies Depression Scale. A self report depression scale for research in the general population. Appl Psychol Measurement.

[10] Wakefield CE, Butow PN, Aaronson NA, Hack TF, Hulbert-Williams NJ, Jacobsen PB (2015). Patient-reported depression measures in cancer: a meta-review. Lancet Psychiatry.

[11] Kroenke K, Spitzer RL, Williams JBW (2001). The PHQ-9 - Validity of a brief depression severity measure. J Gen Intern Med..

[12] Martin A, Rief W, Klaiberg A, Braehler E (2006). Validity of the Brief Patient Health Questionnaire Mood Scale (PHQ-9) in the general population. Gen Hosp Psychiatry.

[13] Thekkumpurath P, Walker J, Butcher I, Hodges L, Kleiboer A, O’Connor M (2011). Screening for major depression in cancer outpatients. The diagnostic accuracy of the 9-item Patient Health Questionnaire. Cancer.

[14] Wittkampf KA, Naeije L, Schene AH, Huyser J, Van Weert HC (2007). Diagnostic accuracy of the mood module of the Patient Health Questionnaire: a systematic review. Gen Hosp Psychiatry.

[15] Watzke B, Heddaeus D, Steinmann M, Koenig H-H, Wegscheider K, Schulz H (2014). Effectiveness and cost-effectiveness of a guideline-based stepped care model for patients with depression: study protocol of a cluster-randomized controlled trial in routine care. BMC Psychiatry.

[16] Kocalevent RD, Hinz A, Brähler E (2013). Standardization of the depression screener patient health questionnaire (PHQ-9) in the general population. Gen Hosp Psychiatry.

[17] Hawley ST, Lantz PM, Janz NK, Salem B, Morrow M, Schwartz K (2007). Factors associated with patient involvement in surgical treatment decision making for breast cancer. Patient Educ Counsel.

[18] Wahl I, Löwe B, Bjorner JB, Fischer F, Langs G, Voderholzer U (2014). Standardization of depression measurement: a common metric was developed for 11 self-report depression measures. J Clin Epidemiol.

[19] Forkmann T, Gauggel S, Spangenberg L, Brahler E, Glaesmer H (2013). Dimensional assessment of depressive severity in the elderly general population: Psychometric evaluation of the PHQ-9 using Rasch Analysis. J Affect Disord.

[20] Kendel F, Wirtz M, Dunkel A, Lehmkuhl E, Hetzer R, Regitz-Zagrosek V (2010). Screening for depression: Rasch analysis of the dimensional structure of the PHQ-9 and the HADS-D. J Affect Disord.

[21] Chilcot J, Rayner L, Lee W, Price A, Goodwin L, Monroe B (2013). The factor structure of the PHQ-9 in palliative care. J Psychosom Res.

[22] Richardson EJ, Richards JS (2008). Factor structure of the PHQ-9 screen for depression across time since injury among persons with spinal cord injury. Rehabil Psychol.

[23] Elhai JD, Contractor AA, Tamburrino M, Fine TH, Prescott MR, Shirley E (2012). The factor structure of major depression symptoms: A test of four competing models using the Patient Health Questionnaire-9. Psychiatry Res.

[24] Krause JS, Bombardier C, Carter RE (2008). Assessment of depressive symptoms during inpatient rehabilitation for spinal cord injury: Is there an underlying somatic factor when using the PHQ?. Rehabil Psychol.

[25] Krause JS, Reed KS, McArdle JJ (2010). Factor structure and predictive validity of somatic and nonsomatic symptoms from the Patient Health Questionnaire-9: A longitudinal study after spinal cord injury. Arch Phys Med Rehabil.

[26] Stafford L, Judd F, Gibson P, Komiti A, Quinn M, Mann GB (2014). Comparison of the Hospital Anxiety and Depression Scale and the Center for Epidemiological Studies Depression Scale for detecting depression in women with breast or gynecologic cancer. Gen Hosp Psychiatry.

[27] Hinz A, Krauss O, Stolzenburg J, Schwalenberg T, Michalski D, Schwarz R (2009). Anxiety and depression in patients with prostate cancer and other urogenital cancer: A longitudinal study. Urol Oncol Semin Ori.

[28] Hjermstad MJ, Fayers PM, Bjordal K, Kaasa S (1998). Using reference data on quality of life-the importance of adjusting for age and gender, exemplified by the EORTC QLQ-C30 (+3). Eur J Cancer.

[29] Aaronson NK, Ahmedzai S, Bergman B, Bullinger M, Cull A, Duez NJ (1993). The European-Organization-For-Research-And-Treatment-Of-Cancer QLQ-C30 - A quality-of-life instrument for use in international clinical trials in oncology. J Natl Cancer Inst.

[30] Herschbach P, Berg P, Dankert A, Duran G, Engst-Hastreiter U, Waadt S (2005). Fear of progression in chronic diseases-Psychometric properties of the Fear of Progression Questionnaire. J Psychosom Res.

[31] Spitzer RL, Kroenke K, Williams JBW, Löwe B (2006). A brief measure for assessing generalized anxiety disorder - The GAD-7. Arch Int Med..

[32] Löwe B, Wahl I, Rose M, Spitzer C, Glaesmer H, Wingenfeld K (2010). A 4-item measure of depression and anxiety: Validation and standardization of the Patient Health Questionnaire-4 (PHQ-4) in the general population. J Affect Disord.

[33] Cohen J (1988). **Statistical power analysis for the behavioral sciences**, ed 2.

[34] Hu LT, Bentler PM (1999). Cutoff criteria for fit indexes in covariance structure analysis: conventional criteria versus new alternatives. Struct Equ Modeling.

[35] Steiger JH (1990). Structural model evaluation and modification - an interval estimation approach. Multivariate Behav Res.

[36] Bentler PM, Bonett DG (1980). Significance tests and goodness of fit in the analysis of covariance structures. Psychol Bull.

[37] Tucker LR, Lewis C (1973). Reliability coefficient for maximum likelihood factor analysis. Psychometrika.

[38] Hammash MH, Hall LA, Lennie TA, Heo S, Chung ML, Lee KS (2013). Psychometrics of the PHQ-9 as a measure of depressive symptoms in patients with heart failure. Eur J Cardiovasc Nurs.

[39] Johns SA, Kroenke K, Krebs EE, Theobald DE, Wu JW, Tu WZ (2013). Longitudinal comparison of three depression measures in adult cancer patients. J Pain Symptom Manage.

[40] Romppel M, Brähler E, Roth M, Glaesmer H (2013). What is the General Health Questionnaire-12 assessing? Dimensionality and psychometric properties of the General Health Questionnaire-12 in a large scale German population sample. Compr Psychiatry.

[41] Walker J, Hansen CH, Butcher I, Sharma N, Wall L, Murray G (2011). Thoughts of death and suicide reported by cancer patients who endorsed the “Suicidal thoughts” item of the PHQ-9 during routine screening for depression. Psychosomatics.

[42] Singer S, Lincke T, Gamper E, Bhaskaran K, Schreiber S, Hinz A (2012). Quality of life in patients with thyroid cancer compared with the general population. Thyroid.

[43] Mehnert A, Berg P, Henrich G, Herschbach P (2009). Fear of cancer progression and cancer-related intrusive cognitions in breast cancer survivors. Psycho-Oncology.

